# Transitions between explosive and effusive phases during the cataclysmic 2010 eruption of Merapi volcano, Java, Indonesia

**DOI:** 10.1007/s00445-016-1046-z

**Published:** 2016-07-18

**Authors:** Katie Preece, Ralf Gertisser, Jenni Barclay, Sylvain J. Charbonnier, Jean-Christophe Komorowski, Richard A. Herd

**Affiliations:** 1 School of Environmental Sciences, University of East Anglia, Norwich, NR4 7TJ UK; 2 School of Physical and Geographical Sciences, Keele University, Keele, Staffordshire ST5 5BG UK; 3 School of Geosciences, University of South Florida, Tampa, FL 33620-5201 USA; 4 Equipe Systèmes Volcaniques, Institut de Physique du Globe de Paris, Sorbonne Paris Cité, Université Paris Diderot, UMR CNRS 7154, 1 rue Jussieu, Paris, 75238 Cedex 05 France

**Keywords:** Microlites, CSD, Merapi, Explosive eruption, Effusive eruption

## Abstract

Transitions between explosive and effusive activity are commonly observed during dome-forming eruptions and may be linked to factors such as magma influx, ascent rate and degassing. However, the interplay between these factors is complex and the resulting eruptive behaviour often unpredictable. This paper focuses on the driving forces behind the explosive and effusive activity during the well-documented 2010 eruption of Merapi, the volcano’s largest eruption since 1872. Time-controlled samples were collected from the 2010 deposits, linked to eruption stage and style of activity. These include scoria and pumice from the initial explosions, dense and scoriaceous dome samples formed via effusive activity, as well as scoria and pumice samples deposited during subplinian column collapse. Quantitative textural analysis of groundmass feldspar microlites, including measurements of areal number density, mean microlite size, crystal aspect ratio, groundmass crystallinity and crystal size distribution analysis, reveal that shallow pre- and syn-eruptive magmatic processes acted to govern the changing behaviour during the eruption. High-An (up to ∼80 mol% An) microlites from early erupted samples reveal that the eruption was likely preceded by an influx of hotter or more mafic magma. Transitions between explosive and effusive activity in 2010 were driven primarily by the dynamics of magma ascent in the conduit, with degassing and crystallisation acting via feedback mechanisms, resulting in cycles of effusive and explosive activity. Explosivity during the 2010 eruption was enhanced by the presence of a ‘plug’ of cooled magma within the shallow magma plumbing system, which acted to hinder degassing, leading to overpressure prior to initial explosive activity.

**Electronic supplementary material:**

The online version of this article (doi:10.1007/s00445-016-1046-z) contains supplementary material, which is available to authorized users.

## Introduction

Transitions between explosive and effusive activity are commonly observed at many subduction zone volcanoes. Worldwide, it has been estimated that 95 % of dome eruptions are associated with an explosive component (Newhall and Melson 1983; Ogburn et al. 2015). Dome-forming eruptions have transitioned between effusive behaviour and explosive Vulcanian and/or (sub)Plinian activity at Mount St. Helens, Montserrat, Pinatubo and Guagua Pichincha to name just a few (e.g. Hammer et al. 1999; Cashman and McConnell 2005; Clarke et al. 2007; Wright et al. 2007; Komorowski et al. 2010). Understanding the driving forces behind eruptive style and factors that influence changes in activity is therefore crucial for hazard assessment and monitoring efforts. However, a complex interplay of factors acts in each system to determine eruptive style, with previous results often reaching seemingly paradoxical conclusions regarding how these factors influence eruption dynamics. For example, recharge with hotter magma has previously been linked to remobilisation of magma, triggering and intensifying the onset of eruption and affecting ascent rate at volcanoes such as Soufrière Hills, Montserrat (Murphy et al. 2000) and El Reventador (Ridolfi et al. 2008). In contrast, at Quizapu, Chile (Ruprecht and Bachmann 2010) and Mount Hood, USA (Koleszar et al. 2012), recharge with hotter magma has been linked to a decrease in explosivity, via the reduction of bulk viscosity, which facilitates degassing and inhibits fragmentation. Magmatic influx to a reservoir may in turn affect magma ascent dynamics, influencing both the rate of magma ascent and whether ascent is sustained or pulsatory in its nature (Wolf and Eichelberger 1997; Scandone et al. 2007). Magma ascent rate governs, and is governed by, volatile exsolution during decompression, with fast ascent often linked to closed-system degassing, resulting in increased potential for explosive activity, and slower ascent often linked to open-system degassing and effusive activity (e.g. Jaupart and Allègre 1991; Villemant et al. 2008). At shallow depths, degassing and crystallisation of groundmass microlites influence magma rheology and may also contribute to regulating the style of eruption (e.g. Melnik and Sparks 1999, 2005; Barmin et al. 2002; Degruyter et al. 2012). Extensive, shallow-level, degassing-induced crystallisation can effectively seal the conduit, resulting in pressure build-up and an explosive eruption, if pressure exceeds a critical threshold related to magma strength (e.g. Stix et al. 1993; Sparks 1997; Melnik and Sparks 1999, 2005; Clarke et al. 2007; Wright et al. 2007; Komorowski et al. 2010; Burgisser et al. 2011). Therefore, a complex interplay between deep and shallow processes related to magma storage, influx, ascent and the rheological changes caused by crystallisation and degassing is known to influence the final eruptive behaviour, often via complex feedback mechanisms.

This paper investigates what caused transitions between explosive and effusive activity during the VEI 4 eruption of Merapi volcano (Indonesia) in 2010, via textural and compositional analysis of feldspar groundmass microlites. Previous studies have used textural and compositional analyses of experimental and natural samples to shed light on the processes and consequences of devolatilisation and crystallisation of groundmass microlites during magma ascent (e.g. Cashman 1988, 1992; Hammer et al. 1999, 2000; Hammer and Rutherford 2002). However, most studies of experimental and natural samples have focussed on a particular eruptive behaviour of a volcano, with comparatively less attention paid to the transitions between eruptive styles (Cashman and McConnell 2005; Castro and Gardener 2008; Martel 2012). The physical changes experienced by magma during ascent have been interpreted via analysis of the textures and compositions of microlites that have grown in response to those changes. Differences in eruptive style have been attributed to magma supply rate (Cashman and McConnell 2005), as well as exsolved volatile pressure in the conduit (Castro and Gardner 2008). At Merapi, investigation of lava samples from the 1986–1995 domes, indicated a correlation between effusion rate and microlite number density, with similar microlite textures throughout that period, suggesting crystallisation conditions are cyclic (Hammer et al. 2000). A detailed study of microlites in lava dome samples extruded throughout the 2006 Merapi eruption revealed that factors such as magma ascent path, source depth and near-surface degassing conditions are recorded in the final groundmass texture of Merapi rocks (Preece et al. 2013). New insights into the driving forces behind transitions in eruptive style can be gained by careful textural analysis of chronologically controlled natural samples linked to a well-documented eruption. In this paper, a well-constrained sample suite representing different stages of the 2010 eruption is linked to eruption chronology, allowing the petrological and textural features to be interpreted in light of eruptive style and monitoring data. In particular, shallow magmatic processes that occur in the edifice and conduit are investigated via quantitative textural and compositional analysis of feldspar microlites. We hypothesise that initiation and cessation of the 2010 eruption were predominantly driven by magma flux at depth, with transitions between explosive and effusive activity in 2010 primarily controlled by the dynamics of magma ascent in the shallow conduit. Degassing and degassing-induced crystallisation during magma ascent in the conduit played a crucial role in influencing eruptive behaviour, via complex feedback mechanisms resulting in cycles of explosive and effusive activity. The 2010 eruption was the largest at Merapi since 1872, and hence, it is the first time that a larger than ‘normal’ eruption has been well-monitored at Merapi, with seismic, ground deformation and gas emission data available (Surono et al. 2012; Budi-Santoso et al. 2013). This eruption therefore provides an ideal case study in which to examine the driving forces behind sudden transitions in eruptive styles during a single, relatively short-lived eruption.

## 2010 eruption chronology, deposits and sampling

### Eruption chronology

During the latter part of the twentieth century and early twenty-first century, eruptions at Merapi have mainly consisted of effusive lava dome growth and subsequent gravitational collapse to generate small volume pyroclastic density currents (PDCs) (VEI 1–3). In stark contrast, the 2010 eruption (VEI 4) began with explosions rather than the effusion of a lava dome and displayed remarkably high extrusion rates (up to 35 m^3^ s^−1^) when dome growth took place subsequently (Surono et al. 2012; Pallister et al. 2013; Ratdomopurbo et al. 2013). Activity in 2010 included several explosive stages generating a series of laterally directed dome explosions (‘blasts’), a subplinian eruption column and maximum PDC runout distances of ∼16 km, more than twice those generated in the effusive dome-forming eruption in 2006 (Charbonnier and Gertisser 2008; Charbonnier et al. 2013; Komorowski et al. 2013).

**Fig. 1:**
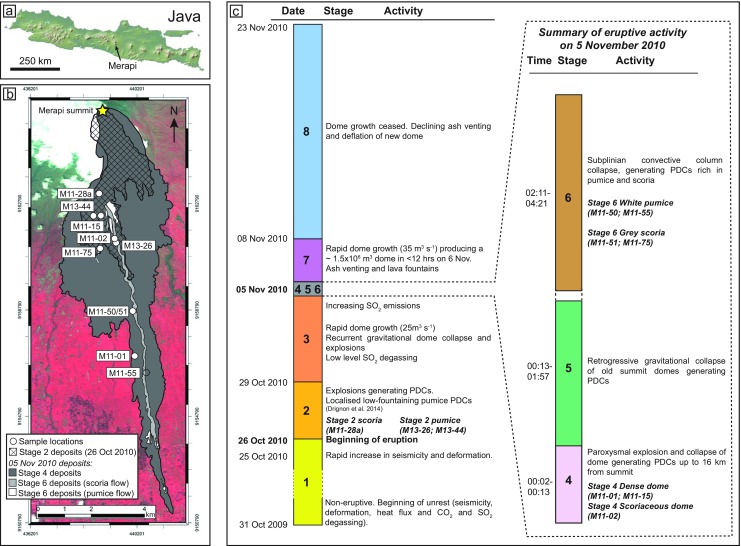
**a** Map of Java, showing the locality of Merapi volcano. **b** Map of localities for samples used in textural analysis and the distribution of the deposits erupted on 26 October (stage 2) and on 5 November (stages 4 and 6) plotted onto a shaded relief DEM (after Komorowski et al. 2013). Map coordinate system is WGS 84 with decimal degrees. **c** Eruptive timeline of the 2010 eruption, with stages based on Komorowski et al. (2013) showing expanded section describing the paroxysmal eruptive activity of 5 November and showing samples used for textural analysis

The 2010 eruption chronology (Fig. 1) has previously been documented in detail (e.g. Surono et al. 2012; Pallister et al. 2013; Charbonnier et al. 2013; Komorowski et al. 2013). Komorowski et al. (2013) recognised eight stages of the 2010 eruption, which will be referred to throughout this paper: stage 1 (31 October 2009–26 October 2010), unrest and magmatic intrusion; stage 2 (26 October 2010), initial explosions; stage 3 (29 October–4 November 2010), recurrent rapid dome growth (up to 25 m^3^ s^−1^) and destruction; stage 4 (5 November 2010), paroxysmal dome explosions and collapse; stage 5 (5 November 2010), retrogressive summit collapse; stage 6 (5 November 2010), subplinian convective fountain collapse; stage 7 (5–8 November 2010), rapid dome growth (35 m^3^ s^−1^) with alternating effusive and explosive activity; and stage 8 (8–23 November 2010), declining ash venting and degassing.

### Eruption deposits and sampling

The 2010 eruption deposits were reported in detail by Charbonnier et al. (2013), Cronin et al. (2013), Komorowski et al. (2013) and Preece (2014). The deposits are briefly described and documented here with the aim of relating petrological and textural analysis to eruption chronology and dynamics.

Stage 1 is defined as a period of unrest and magmatic intrusion prior to eruption. Therefore, no deposits or samples are associated with stage 1.

The first deposits of the 2010 eruption were produced during explosive activity on 26 October. These mark the onset of the eruption and the beginning of stage 2. Stage 2 deposits consist of dilute PDC (surge) deposits (Komorowski et al. 2013), valley-confined block-and-ash flows (BAFs) (Charbonnier et al. 2013) and pumice-rich PDCs (Drignon et al. 2014) (Fig. 1). Typically, each surge unit consists of two layers; the lower layer composed of massive, grey-coloured or ‘salt and pepper’ coarse ash to fine lapilli. The upper layer is usually a brown-orange or grey-coloured fine ash, which is often stratified. Componentry analysis of clasts and matrix (Charbonnier et al. 2013) reveals that the dominant clast component (∼90 %) of the stage 2 deposits is fresh, vesicular scoriaceous material (herein referred to as ‘stage 2 scoria’). Stage 2 deposits also contain conspicuous juvenile white pumice clasts (‘stage 2 pumice’), light-coloured dense fragments as well as non-juvenile, hydrothermally altered and accidental lithics (see Online Resource 1 for representative logs).

Stage 3 consisted of repeated, rapid (up to 25 m^3^ s^−1^) dome growth and recurrent destruction by explosions and collapses, emplacing dilute PDC (surge) deposits (Komorowski et al. 2013) and valley-confined BAFs (Charbonnier et al. 2013).

The paroxysmal stage 4 activity consisted of five laterally directed dome explosions over a period of 11 min which destroyed the rapidly emplaced lava dome (Komorowski et al. 2013). Stage 4 deposits, formed during these cataclysmic dome explosions, include valley-confined BAFs and associated overbank deposits which were generated via the breakout of confined flows onto interfluve areas, as documented by Charbonnier et al. (2013), as well as high-energy, dilute PDC (surge) or ‘blast’ deposits (Komorowski et al. 2013) (Fig. 1). The total volume of the 2010 deposits is ∼36.3 × 10^6^ m^3^, with >70 % of this volume deposited during stage 4 (Charbonnier et al. 2013). Field observations, componentry analysis (Charbonnier et al. 2013) and clast density measurements (Komorowski et al. 2013) reveal bimodal dome density and vesicularity. Stage 4 deposits are essentially monolithological, in that their componentry is dominated (>80 %) by dark grey to black, dense (∼2.5 g cm^−3^; Komorowski et al. 2013) fragments of the fast-growing dome, which was destroyed by the stage 4 explosions (herein referred to as ‘stage 4 dense dome’ clasts). As well as these dense dome fragments, dark grey to black scoriaceous dome fragments also occur within stage 4 deposits (herein referred to as ‘stage 4 scoriaceous dome’ clasts). This lower density (as low as 1.9 g cm^−3^; Komorowski et al. 2013) scoriaceous dome material reflects the existence of dome regions that had not entirely degassed and produced vesicular juvenile dome fragments. Light grey, dense, crystalline material (similar to the light grey, dense lithology found in stage 2) is found as abundant angular inclusions ranging in size from millimetres to centimetres within the juvenile dome material (herein referred to as ‘light grey inclusions’). Occasionally, this lithology also forms wavy, diffuse bands through the darker dome material. Large, and in some cases, prismatically jointed blocks (up to several metres in diameter) of this inclusion material have also been found loose within the stage 4 PDC deposits (Online Resource 1).

Retrogressive summit dome collapse (Komorowski et al. 2013) during stage 5 (Fig. 1) produced BAFs consisting of variable lithologies, including older, non-juvenile dome clasts, dense and scoriaceous 2010 dome clasts, light grey dense clasts as well as variable non-juvenile lithics. The deposits often have a distinctive reddish-pink colour. The eruptive lull at the end of stage 5 allowed for the deposition of a layer consisting of orange-pink-coloured fine ash with abundant accretionary lapilli, forming a distinctive marker horizon across Merapi’s southern flanks (Online Resource 1).

Stage 6 activity consisted of ash venting and recurrent subplinian convective column collapse, generating PDCs. Stage 6 deposits related to convective column collapse are rich in scoriaceous or pumiceous clasts (Fig. 1 and Online Resource 1). In particular, a stage 6 PDC rich in grey scoriaceous clasts (herein referred to as ‘stage 6 grey scoria’) was sampled in the Kali Gendol and on the Kali Gendol interfluve areas (Fig. 1). Scattered on the surface of the scoria-rich flow deposits, abundant white pumice clasts occur (herein referred to as ‘stage 6 white pumice’) that are also interpreted to be associated with the stage 6 column collapse events (Komorowski et al. 2013). Clast density of pumice is 1.25–1.48 ± 0.2 g cm^−3^ (Komorowski et al. 2013).

The 2010 deposits were characterised during three field seasons (February 2011, July 2011, August 2013). Stratigraphic logs (Online Resource 1) and componentry analysis (Charbonnier et al. 2013) enabled units and lithology types to be linked to the eruptive stages of Komorowski et al. (2013) (Fig. 1). Based on field observations and componentry, >200 samples were collected from logged sites, related to eruption stage and various style of activity. To assess inter-clast chemical variability, samples were analysed by XRF to determine whole-rock major element compositions and by electron microprobe to determine mineral and glass compositions (see Online Resource 2 for samples analysed and Preece (2014) for full sample dataset). Whole-rock compositions are similar for all analysed 2010 juvenile material (54.7–55.7 wt% SiO_2_), with light grey inclusion material being slightly less evolved (52.6–54.4 wt% SiO_2_) (Online Resource 3). Juvenile whole-rock compositions are similar to the effusive 2006 eruption (Preece et al. 2013), as well as previous dome-forming eruptions (Gertisser et al. 2012). To assess inter-clast textural variability, ∼50 thin sections were studied, with a sub-set of these samples chosen for feldspar microlite textural and compositional analysis, including stage 2 scoria and pumice produced during the initial explosions on 26 October, stage 4 dense and scoriaceous samples from the rapidly grown and destroyed stage 3 lava dome, light grey inclusion material recovered from stage 4 PDC deposits, interpreted to be derived from a pre-eruption conduit plug (Preece 2014; Gertisser et al. 2015) as well as grey scoria and white pumice from stage 6 subplinian column collapse. This analysis therefore provides the means to compare groundmass microlite textures produced via effusive and explosive activity that occurred during the main stages of the 2010 eruption.

## Methods

Mineral and glass major and minor element compositions were determined using Cameca SX-100 electron microprobes at the University of Cambridge, The Open University and the University of Edinburgh. Feldspar microlites were analysed using a beam diameter of 1, 5 or 10 μm, a 15–20-kV accelerating voltage and a 10–20 nA beam current. Groundmass glass was analysed using a 5–10-μm beam size, a 15-kV accelerating voltage and a 4–10-nA beam current. Na was always counted first to minimise alkali migration. Natural silicate minerals were used as primary standards to calibrate instruments and as secondary in-run standards to monitor precision and accuracy during mineral analyses. Rhyolitic glasses, including KE-12, a peralkaline obsidian from Kenya, were used as secondary in-run standards to monitor glass analyses. Detection limits were ∼100–500 ppm for the elements analysed and analytical uncertainties, based on repeat analyses of natural mineral and rhyolitic glass standards, were ∼1–3 % (relative).

Textural analysis of feldspar microlites was carried out using the methods of Preece et al. (2013). In brief, multiple back-scattered electron (BSE) images of continuous areas of groundmass were acquired with a JEOL JSM 5900 LV scanning electron microscope (SEM) at the University of East Anglia, using an accelerating voltage of 20 kV and a working distance of 10 mm at ×1000 or ×1500 magnification. Images were digitally stitched together, and a minimum of ∼600 feldspar microlites in each sample were outlined manually using *Adobe Illustrator*. Measurements of feldspar microlite size and abundance, sample vesicularity and the area occupied by other mineral phases (i.e. pyroxene, Fe-Ti oxide and apatite microlites) were made using *ImageJ*. Crystal length and width measurements were converted to 3D crystal habits using *CSDslice* (Morgan and Jerram 2006) to produce a characteristic aspect ratio (short, *S*; intermediate, *I*; long axis, *L*) for each sample. *CSDCorrections* (*version 1.39*) (Higgins 2000) was utilised to calculate crystal size distributions (CSDs), using the ellipsoid minor axis for samples with acicular crystals and the ellipsoid major axis for those with rectangular prism aspect ratios, as recommended in *CSDCorrections* as it reflects the most likely 2D intersections for these shapes. For all samples, the rock fabric was massive and the crystal aspect ratio calculated with *CSDslice* was used. Crystal roundness was estimated to be 0.1 (on a scale from 0 to 1, where 0 is a euhedral block and 1 is an ellipsoid). The area percent of vesicles was used to correct for sample vesicularity in the calculation of crystal population density. The number of logarithmic size intervals, or bins, was set at five per decade so that each bin is 1.6 times the size of the next smallest bin, and any bins containing less than five crystals were removed from the CSD as they are not precise, as suggested by Higgins (2000).

## Results

### Groundmass feldspar microlite textures and compositions

**Fig. 2:**
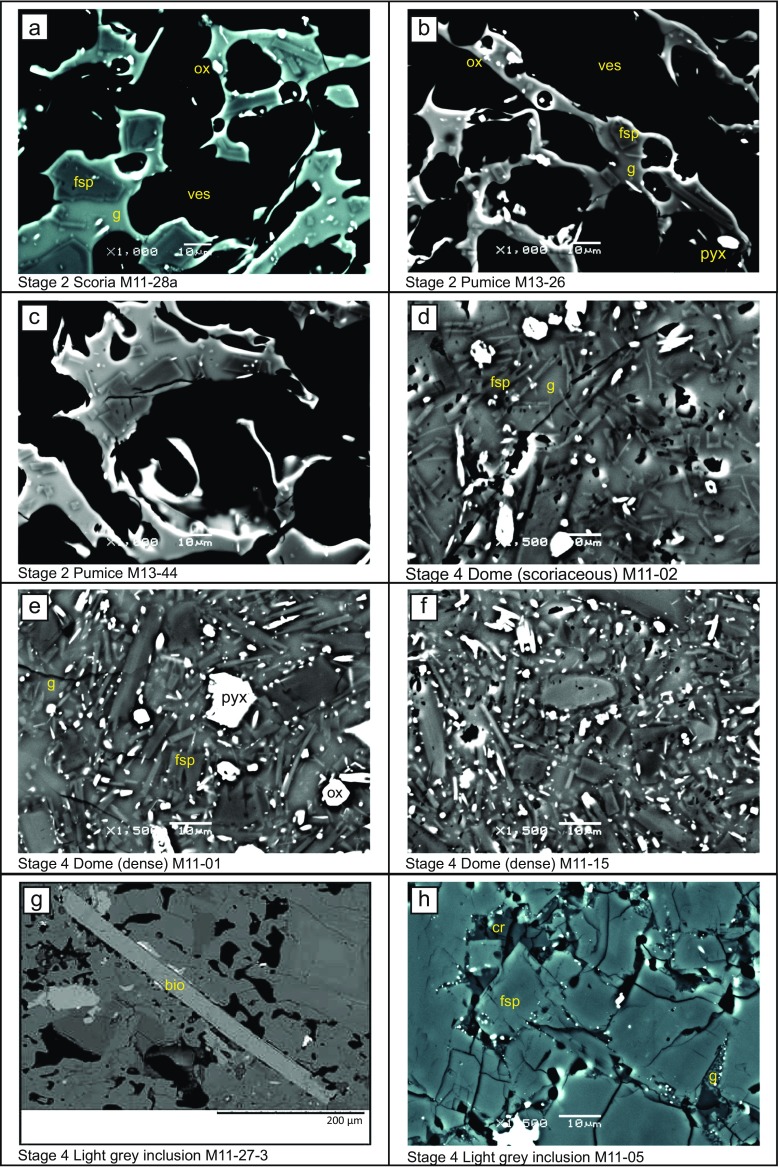

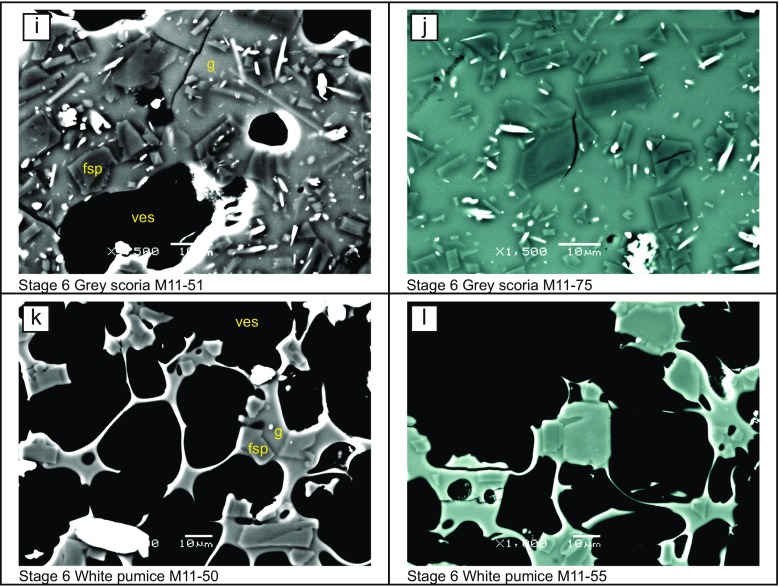
**a**–**l** Representative BSE images of 2010 lithologies showing feldspar microlites (*fsp*), pyroxene microlites (*pyx*), Fe-Ti oxide microlites (*ox*), groundmass glass (*g*) and vesicles (*ves*). Note the presence of biotite (*bio*) and cristobalite (*cr*) and the speckled appearance of the groundmass in the light grey inclusion material. All images were taken at ×1500 magnification except those of stage 2 and stage 6 pumice samples taken at ×1000 magnification and **g** taken at ×400 magnification

**Fig. 3:**
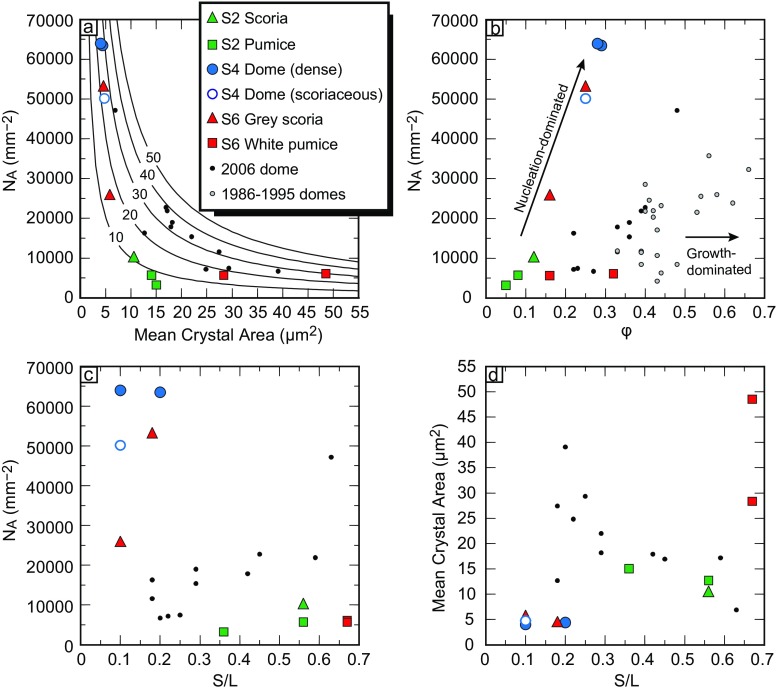
Variation in textural parameters in 2010 products, plotted with those from 2006 dome products (Preece et al. 2013) for comparison. **a** Areal feldspar microlite number density (*N*_A_ mm^−2^) vs. mean crystal area (μm^2^), with *solid lines* indicating modelled microlite crystallinity at a particular *N*_A_ and crystal size, labelled with percentage values. **b** Areal feldspar microlite number density (*N*_A_ mm^−2^) vs. feldspar microlite crystallinity (*φ*) including data from other recent (1986–1995) effusive dome-forming eruptions (Hammer et al. 2000). **c** Areal feldspar microlite number density (*N*_A_ mm^−2^) vs. short/long axis (*S*/*L*). **d** Mean crystal area (μm^2^) vs. *S*/*L*

Visual inspection of the groundmass in texturally representative samples reveals clear differences between each lithology type (Fig. 2). Textural analysis of feldspar microlites, the most abundant groundmass phase, reveals variations in crystal size, population density and morphology between different lithologies (Fig. 3 and Table 1). Microlite abundance, expressed as areal number density (*N*_A_), describes the number of crystals per unit area (mm^2^) on a vesicle- and phenocryst-free basis. The highest number densities are in samples from the stage 4 dome, especially the dense dome samples (up to 63,962 mm^−2^). The lowest number densities are recorded in samples of stage 2 and 6 pumice (minimum 3230 mm^−2^). For most lithologies, mean crystal area ranges between ∼4–15 μm^2^, although the average crystal size microlites in the white pumice samples is larger (28 and 49 μm^2^). Feldspar microlite crystallinity (*φ*) is the fraction of groundmass area that is occupied by feldspar microlites and, following the calculations of Hammer et al. (2000), is expressed on a vesicle-, phenocryst- and other crystal phase-free area, meaning that the remaining area is composed of only groundmass glass, thereby taking into account the area of liquid available for late-stage crystallisation. It is a function of both microlite abundance and size, as many small crystals may produce the same crystallinity as few large crystals. Feldspar microlite crystallinity ranges from 0.05 to 0.32 in 2010 samples (Table 1). The morphology of the crystals calculated using *CSDSlice* (Morgan and Jerram 2006) is generally revealed to be acicular, apart from those in the stage 2 scoria and the stage 6 white pumice, which are classed as having a rectangular prism habit. Short/long (*S*/*L*) axis ratios range from 0.10 to 0.67, with acicular shaped crystals having a low *S*/*L* and more blocky shaped crystals having a higher *S*/*L*. The stage 6 white pumice microlites have the highest *S*/*L*, with both sampled populations calculated at 0.67 (Fig. 3). In summary, the 2010 products have either many small microlites (e.g. stage 4 dense dome) or few large microlites, such as in the stage 6 white pumice samples. The relatively few and large microlites of the white pumice have blocky morphologies, whereas the abundant small microlites in the dome samples are acicular in shape. Light grey inclusion material has a highly crystalline groundmass, which has not been analysed quantitatively because many microlites are touching, making discrimination of separate crystals difficult (Fig. 2g, h). However, qualitative observations show that the highly crystalline groundmass is composed of tabular or equant looking crystals, with low proportions of groundmass glass. The remaining glass often appears speckled, with heterogeneities of light and dark areas in backscatter images. Within the light grey inclusions, biotite (Fig. 2g) and a crystalline SiO_2_ phase, cristobalite, are also present (Fig. 2h). Platy cristobalite crystals fill small vesicles and are pervasive within the groundmass, often with ‘fish-scale’ cracked morphology or a microbotryoidal texture. Biotite has not previously been observed in Merapi products before 2010. Costa et al. (2013) note the presence of biotite in 2010 samples, with Preece (2014) proposing its presence is predominantly restricted to the light grey inclusion material.

**Table 1:** Textural variation of 2010 feldspar microlites

Stage	Lithology	Sample	2D areal measurements									3D volumetric measurements		
			*n* ^a^	Total area (μm^2^)	*N*_A_ (mm^−2^)	Area % fsp (vesicle corrected)	φ	Mean crystal area (μm^2^)	Groundmass glass (area %)	Vesicle area %	*S*/*L*	Calculated aspect ratio (*R* ^2^)	Nv (mm^−3^)	Vol. from inter-area (%)
2	S2S	M11-28a	716	265,942	10,396	11.0	0.12	10.58	81.38	74.8	0.56	1.00:1.25:1.80 (0.8883)	3,050,000	11.3
2	S2P	M13-26	696	375,000	5704	8.2	0.08	14.63	89.34	66.14	0.56	1.00:1.50:1.80 (0.7626)	1,740,000	7.9
2	S2P	M13-44	604	406,000	3230	4.8	0.05	15.05	89.30	54.48	0.36	1.00:1.40:2.80 (0.7546)	1,060,000	4.9
4	DD	M11-15	678	10,682	63,471	27.8	0.29	4.40	67.73	N/A	0.20	1.00:1.60:5.00 (0.7245)	17,500,000	27.9
4	DD	M11-01	790	12,351	63,962	25.8	0.28	4.03	66.43	N/A	0.10	1.00:1.50:10.00 (0.6928)	8,880,000	25.7
4	SD	M11-02	736	15,612	50,150	23.9	0.25	4.76	70.70	6.0	0.10	1.00:3.20:10.00 (0.5101)	10,900,000	23.9
6	GS	M11-75	717	27,568	26,009	15.2	0.16	5.84	82.04	N/A	0.10	1.00:1.50:10.00 (0.7869)	2,850,000	14.4
6	GS	M11-51	779	18,082	53,338	24.5	0.25	4.59	72.58	18.2	0.18	1.00:1.40:5.50 (0.7512)	13,600,000	24.2
6	WP	M11-50	686	330,746	6111	29.6	0.32	48.52	62.97	64.1	0.67	1.00:1.15:1.50 (0.8533)	655,000	28.0
6	WP	M11-55	645	297,422	5653	16.0	0.16	28.35	83.01	59.7	0.67	1.00:1.10:1.50 (0.8407)	853,000	15.2

Lithology types are as follows: *S2S* stage 2 scoria, *S2P* stage 2 pumice, *DD* dome (dense), *SD* dome (scoriaceous), *GS* grey scoria, *WP* white pumice. Area percent feldspar has been vesicle corrected where necessary

^a^Number of crystals analysed

**Fig. 4:**
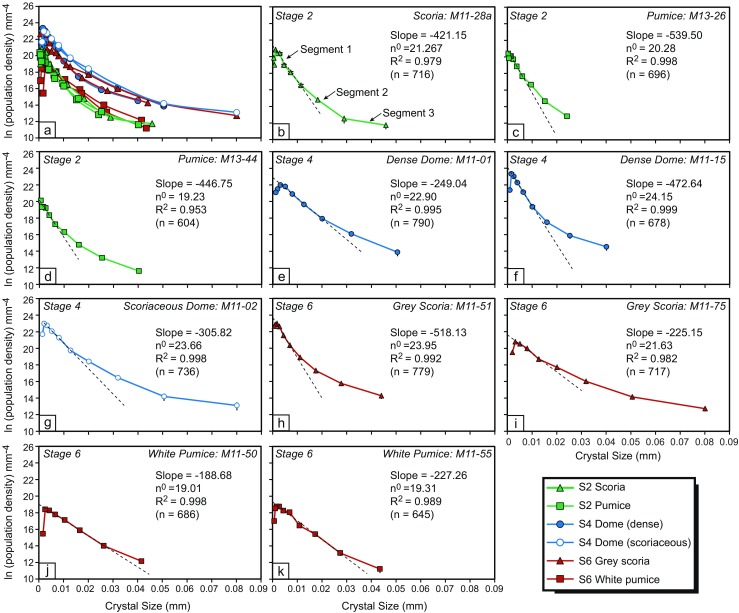
Crystal size distribution (CSD) plots of feldspar microlites from the 2010 eruption. **a** All 2010 CSDs. **b**–**k** Individual CSDs for each sample (*coloured solid line*), with a regression line fitted to the steepest part of the CSD curve (*black dashed line*), values for the slope of the regressed line, the *y*-axis intercept or nucleation density (*n* ^o^) and the *R* ^2^ value. *n* denotes the number of crystals analysed in each sample

The two-dimensional intersection measurements as outlined above were converted into 3D data using *CSDSlice* (Morgan and Jerram 2006) and *CSDCorrections* (Higgins 2000) to produce CSDs, as described in Preece et al. (2013) (Fig. 4). All of the CSDs display curved trends, which may be divided into two or three segments (Fig. 4). The decrease in population density at the smallest sizes, resulting in a left-hand ‘downturn’ is considered to result from conversion errors in the CSD software, resulting from a lower probability of intersecting smaller crystal sizes (Cashman 1988; Brugger and Hammer 2010a). The *y*-axis intercept (*n* ^o^), calculated using the steepest part of the CSD, not including the left-hand downturn, is indicative of the final nucleation density that ranges from 18.84 to 23.37 mm^−4^, with stage 6 white pumice samples having the lowest intercept values and the dense dome fragments having the highest. This is consistent with the *N*_A_ data calculated, which also shows that the dense dome samples have the highest crystal number density and the white pumice has the lowest.

**Fig. 5:**
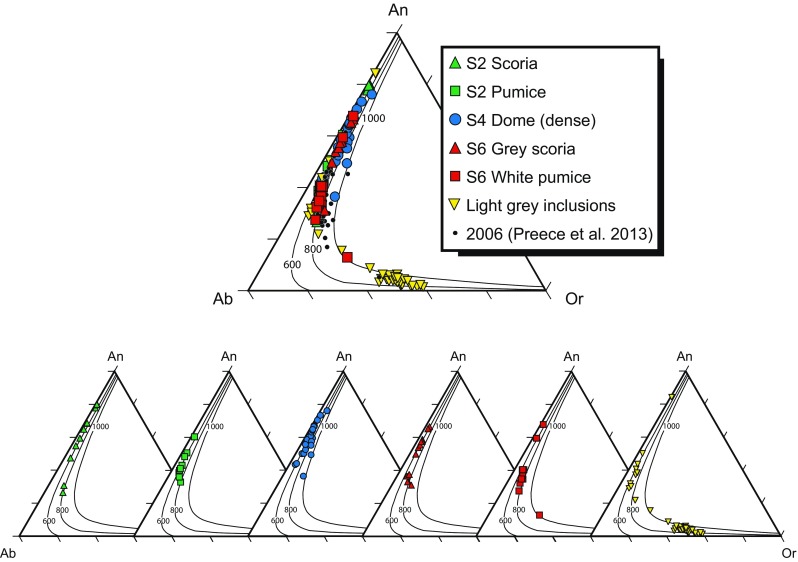
Feldspar microlite compositions plotted in the feldspar ternary (An-Ab-Or) diagram display differences in the compositions between the different 2010 lithologies; 2006 microlites (Preece et al. 2013) are shown for comparison. *Curves* represent isothermal sections of the dry ternary solvus at 600, 800 and 1000 °C, calculated using SOLVCALC (Wen and Nekvasil 1994)

Feldspar microlite compositions (Fig. 5) in the 2010 products are highly variable, ranging from An_1_Ab_41_Or_58_ to An_84_Ab_16_Or_<1_ (Online Resource 4). Microlites from the stage 2 scoria range between An_26_Ab_64_Or_10_ and An_80_Ab_19_Or_<1_ and those from stage 2 pumice between An_33_Ab_59_Or_8_ and An_50_Ab_47_Or_3_. Microlites from dome samples range in composition from An_36_Ab_53_Or_11_ to An_76_Ab_21_Or_3_, although nearly 60 % of the measured microlites have >An_60_ (Fig. 5). Stage 6 grey scoria microlites have a similar overall range in composition between An_31_Ab_59_Or_10_ and An_66_Ab_32_Or_2_, but, in contrast to the dome microlites, only ∼15 % of those from the grey scoria are >An_60_ (Fig. 5). Stage 6 white pumice microlites are generally more albitic (An_12_Ab_61_Or_27_ and An_59_Ab_39_Or_2_), with 90 % of those microlites analysed having ≤ An_40_. Microlites from the light grey inclusion material show the widest overall range (An_1_Ab_41_Or_58_ to An_84_Ab_16_Or_<1_), although more than 70 % of these crystals are alkali feldspars with >Or_20_ (Fig. 5). Microlite compositions are shown with isothermal sections of the dry ternary solvus, calculated using SOLVCALC (Wen and Nekvasil 1994) at 0.5 kbar (Fig. 5). The dry solvus position is not sensitive to pressure changes between 0 and 3 km, the region of expected degassing in the conduit, so depth only controls feldspar composition in terms of the extent of H_2_O degassing (Hammer et al. 2000). The microlite compositions with respect to the dry solvus do not necessarily imply that microlite crystallisation occurred at different temperatures during cooling between ∼600 and 1000 °C but rather that changes in H_2_O activity changed the position of the liquidus and, therefore, the composition of the crystallising feldspar, with enrichment in alkalis due to degassing and the compositional evolution of the residual melt phase.

## Discussion

### Textural and compositional evidence for microlite growth and magma ascent conditions

Groundmass microlite textural differences are due to differences in undercooling (*ΔT*) during crystallisation, which may occur either during ascent or during dome residence. The 2010 dome, which was destroyed on 5 November (stage 4 dense and scoriaceous dome samples), began to extrude on 1 November (Pallister et al. 2013). Therefore, these 2010 dome clasts resided for a maximum of 4 days within the dome. These dome samples have *φ* and groundmass glass area values similar to those of the stage 6 grey scoria and white pumice, which erupted explosively and were never part of a dome. This suggests that these microlite textures predominantly reflect conditions during magma ascent rather than microlite growth during dome residence, as also observed in 2006 dome samples (Preece et al. 2013).

Stage 2 scoria and pumice have low *N*_A_ and *φ* and are closer to intersecting the origin (Fig. 3b), suggesting crystallisation was dominated by nucleation rather than by growth, as also observed in other nucleation-dominated systems (e.g. Pinatubo; Hammer et al. 1999). In stage 4 dome samples, high microlite number densities, small crystal sizes and acicular morphologies also point to nucleation-dominated crystallisation at high *ΔT* (e.g. Lofgren 1980). The extrusion rate of the dome that produced the stage 4 samples was up to 25 m^3^ s^−1^ (Pallister et al. 2013). In comparison, this is nearly 8 times greater than peak extrusion rates at Merapi in 2006 (Ratdomopurbo et al. 2013) and 78 times greater than in 1995 (Hammer et al. 2000). Compared to previous dome eruptions at Merapi, high extrusion rates and high ascent rates of the 2010 dome magma resulted in high degrees of *ΔT* related to degassing during fast decompression in the conduit, causing nucleation-dominated crystallisation and yielding textures with many, small acicular crystals. High ascent rates are also confirmed by the lack of reaction rims on many of the amphibole phenocrysts and microphenocrysts in the dome samples (Preece 2014). Values of *N*_A_ are higher for a given *φ* compared to the 2006 and other recent dome-forming eruptions (Fig. 3b), indicating that crystallisation was dominated by high levels of nucleation in comparison to other recent eruptions. Stage 6 white pumice microlites were presumably also formed during high rates of magma ascent in the subplinian stage of the 5 November paroxysmal activity and yet have different groundmass textures to the dome, with fewer, larger equant microlites (Fig. 3). At very high *ΔT*, often associated with explosive eruptive behaviour, crystallisation is retarded due to an increase in viscosity with H_2_O loss (e.g. Hess and Dingwell 1996) and due to a lag time between conditions of supersaturation and crystallisation. Nucleation kinetics are more sluggish than those of gas exsolution, attributed to the time necessary for structural reorganisation of the melt (Hammer et al. 1999; Brugger and Hammer 2010b), leading to a nucleation delay of between a few hours to days, depending on *ΔT* (Couch et al. 2003a, b; Brugger and Hammer 2010b; Martel 2012). In the stage 6 white pumice samples, nucleation densities are low for a given *φ* compared to other 2010 samples (Fig. 3), indicating that crystallisation proceeded by growth of existing crystals rather than nucleation of new ones. Stage 6 white pumice microlites with large size, low number density and equant morphologies probably crystallised at lower *ΔT* under growth-dominated conditions, before the rapid ascent to the surface. The final rapid ascent did not result in a nucleation event and formation of many small crystals, as seen in the dome samples, because the ascent was sufficiently rapid to hinder crystallisation.

Groundmass microlite textures in stage 6 grey scoria samples are intermediate between those observed in the dome clasts and those in the white pumice, although, in many respects, they are more closely similar to the dome samples and fall on the same nucleation-dominated trend (Fig. 3b). The crystal number densities are more variable than for other lithologies, varying from high number densities, similar to those of the dome, to densities of ∼26,000 mm^−2^ intermediate between those of the dome and white pumice (Fig. 3). Small mean crystal sizes and low aspect ratios reveal that crystals are small and acicular, as in the dome samples. High number densities of small, acicular crystals indicate crystallisation at conditions of high *ΔT*, although not high enough to retard nucleation as recorded by stage 6 white pumice samples. Although quantitative textural analysis was not carried out on the light grey inclusions in the 2010 lava dome, high groundmass crystallinity, low proportions of groundmass glass and the presence of cristobalite suggest that the light grey inclusion material has spent a prolonged period of time crystallising at shallow depths (<50 MPa) within the magmatic system (Couch et al. 2003b). The presence of cristobalite is indicative of extensive late-stage vapour-phase crystallisation as previously observed, for example, in the Mount St. Helens cryptodome in 1980 and subsequent dome rocks (Hoblitt and Harmon 1993; Pallister et al. 2008). The inhomogeneous groundmass glass is similar to the glass observed from Mount St. Helens (Cashman 1988, 1992), Santiaguito (Scott et al. 2012) and Soufrière Hills, Montserrat (Couch et al. 2003b). This texture has been interpreted as a phase separation and devitrification of the glass during shallow storage or slow extrusion (Cashman 1992; Scott 2012). Another groundmass phase observed in the light grey inclusions is biotite. This is extremely rare at Merapi and has not been recorded in previous eruptive products prior to 2010. Biotite may have a magmatic origin but possibly may also originate from hydrothermal alteration of basaltic andesite or may be derived from metasomatic skarn material (e.g. Panigrahi et al. 2008; Afshooni et al. 2013; Balassone et al. 2013). Biotite composition, especially in terms of its FeO, MgO, MnO, TiO_2_ and Al_2_O_3_ contents, is indicative of its mode of formation. For example, the mole fraction of Mg in the octahedral site (*X*_Mg_) has been used to distinguish between igneous biotite and that which formed in hydrothermal alteration zones of porphyry deposits, with the hydrothermal biotite containing higher *X*_Mg_ and less FeO (Selby and Nesbitt 2000; Panigrahi et al. 2008). At Vesuvius for example, distinct mica compositions have been identified, depending on whether they are magmatic or associated with hydrothermal or metasomatic material (Balassone et al. 2013). When compared to the Vesuvius mica compositions, the Merapi biotite are most similar to the biotite of magmatic origin, as they are Al-, Ti- and Fe-rich compared to the skarn and hydrothermal types. As the light grey inclusion material has a highly crystalline groundmass, the biotite potentially formed in the latest magmatic stages from the presumably more evolved residual melt.

In order to further elucidate magmatic ascent conditions, melt H_2_O contents were calculated with the plagioclase-liquid hygrometer of Waters and Lange (2015), using the microlite and groundmass glass compositions (Online Resource 2) and a temperature of 900 °C as inputs. The temperature is based on minimum estimates from amphibole thermometry (Costa et al. 2013; Preece 2014). The range in calculated melt H_2_O contents and the corresponding pressures, calculated using Papale et al. (2006), is as follows: stage 2 scoria, 2.4–3.1 wt% H_2_O (413–624 bars); stage 2 pumice, 2.3–2.6 wt% H_2_O (388–470 bars); stage 6 scoria, 2.1–2.8 wt% H_2_O (334–526 bars); stage 6 pumice, 2.2–3.1 wt% H_2_O (378–642 bars); and light grey inclusions, 1.1–2.3 wt% H_2_O (150–429 bars). Calculations were not performed on stage 4 samples, as groundmass glass patches were too small to analyse accurately. The similar H_2_O content and pressure range for all juvenile 2010 material correspond to microlite crystallisation depths of 1.2–2.4 km, assuming a crustal density of 2800 kg m^−3^ (Costa et al. 2013) and shallower depths of 0.6–1.5 km for the microlites within the light grey inclusions. This suggests that magma decompression and resultant microlite crystallisation originated from similar depths throughout the eruption. However, these results must be taken with some caution, as degassing-induced crystallisation may not occur under full equilibrium conditions.

High-An (up to An_80_) compositions of feldspar microlites in stage 4 dome clasts and stage 2 scoria can result from either higher water pressures or higher temperatures compared to the other, lower An microlites, as experiments show that An content increases with increasing temperature and P(H_2_O) (Couch et al. 2003a; Martel 2012). However, hygrometry results suggest that stage 2 microlites were forming at similar P(H_2_O) compared to all other stages. As such, it is likely that the high-An microlites in the initial explosions and subsequent dome formed via the interaction with an influx of hotter magma, the presence of which is consistent with previous petrological work (Costa et al. 2013; Preece et al. 2014) and monitoring data from the 2010 eruption (Surono et al. 2012). This interpretation is consistent with high-An microlites documented at Mount Pelée (Martel et al. 2006) and Soufrière Hills volcano (Humphreys et al. 2009), which are thought to be inherited from mafic magma. The stage 6 white pumice microlites grew at low *ΔT* under growth-dominated conditions, suggesting the magma stalled in the conduit for a period of time, at depths of ∼1.4–2.4 km. The very fast final ascent and magma fragmentation before the explosive activity only occurred within the last kilometres of ascent, rather than by very fast ascent from greater depths. Therefore, stage 6 may have been caused by rapid decompression and fragmentation driven by unloading, leading to explosive behaviour. Anorthoclase and more K-rich feldspar microlites that are predominant in the light grey inclusion material suggest that crystallisation occurred either at low P(H_2_O) and/or lower temperatures. Hygrometry results are consistent with low P(H_2_O), suggesting crystallisation occurred at depths of 0.6–1.5 km, shallower than other 2010 products and in accord with this material originating from a plug in the shallow conduit (Preece 2014; Gertisser et al. 2015).

### Crystal size distribution analysis

**Fig. 6:**
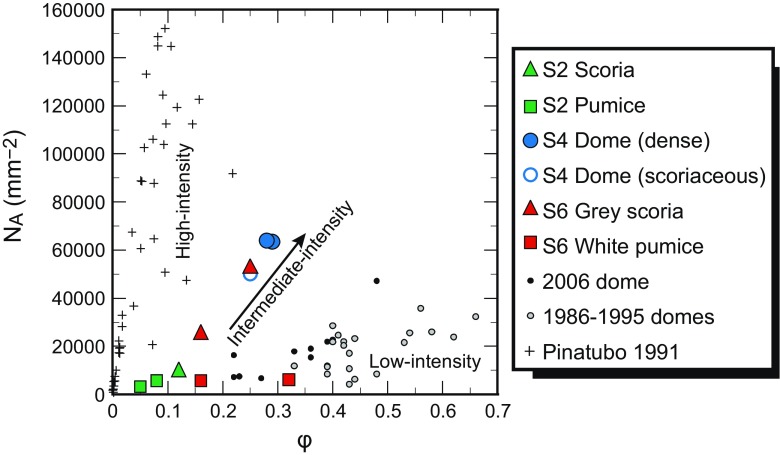
*N*_A_–*φ* relationship of 2010 Merapi samples compared to those from the lower-intensity Merapi 2006 dome-forming eruption (Preece et al. 2013) and older Merapi domes (1986–1995) (Hammer et al. 2000), as well as samples from the higher-intensity pre-climatic stages of the Pinatubo 1991 eruption (Hammer et al. 1999)

Crystal size distributions of the samples are concave-upwards curves, reflecting either shape variability of the sample (Castro et al. 2003) or progressive changes in crystal growth and nucleation rates as a function of changing *ΔT* in the system. Growth of crystals is dominant in the lower conduit, and nucleation begins to dominate in the upper conduit, leading to crystallisation of smaller crystals. Therefore, different microlite sizes reflect magmatic conditions at different depths (Melnik et al. 2011). As crystal growth rate is not constant over time, due to changes in *ΔT* during ascent, a single growth rate cannot be accurately used to solve crystallisation times. However, average crystallisation rates may be calculated using known time constraints based on observations of the eruption chronology. For example, during 1–26 October 2010, an increase in seismic activity and summit deformation marked an ‘intrusive phase’ believed to reflect the movement of magma to shallower (<5 km below the summit) regions in the volcano magma plumbing system (Budi-Santoso et al. 2013), likely inducing microlite crystallisation. Based on this observation, it is possible to calculate an average crystallisation rate for the first microlites grown in the stage 2 scoria and pumice, erupted on 26 October. Using the equation Slope *= −1* / *Gτ*, where *G* is growth rate and *τ* is crystallisation time (Marsh 1988), average crystallisation times can be calculated using the shallowest segment of the CSD (representing the largest microlites) and a crystallisation period of 26 days (Fig. 4). This reveals a crystal growth rate of 9.5 × 10^−9^ mm s^−1^ for the large stage 2 scoria microlites and slower rates of 2.2–3.5 × 10^−9^ mm s^−1^ for stage 2 pumice microlites. These calculated rates are close to the low end of the range of growth rates (10^−6^ to 10^−8^ mm s^−1^) deemed appropriate for syn-eruptive plagioclase crystallisation based on experimental data (Brugger and Hammer 2010b). The fact that the calculated growth rates are at the lower end of this range may be expected for these early microlites that formed deeper within the conduit during growth-dominated crystallisation conditions. Crystals formed later during ascent, at shallower levels within the conduit, may have faster growth rates. The *N*_A_–*φ* relationship (Fig. 6) suggests higher eruption intensity and possibly higher decompression rates, leading to faster growth rates for microlites in stage 4 dome and stage 6 grey scoria samples. In comparison, samples from the 1991 Pinatubo eruption have a higher average feldspar microlite growth rate of 1.7 × 10^−7^ mm s^−1^ (Hammer et al. 1999), consistent with higher eruption intensity compared to Merapi in 2010. This is also in accord with the *N*_A_–*φ* plot (Fig. 6), where the Merapi stage 4 dome and stage 6 grey scoria samples plot within the region between the Pinatubo and previous Merapi effusive dome products, in agreement with the Merapi 2010 eruptive style also being of an intermediate intensity between Pinatubo and previous Merapi effusive dome-forming eruptions.

### Transitions between explosive and effusive eruptive behaviour

The 2010 eruption was the largest at Merapi since 1872 and displayed transitions between both explosive and effusive behaviour. Whole-rock compositions remained constant throughout the duration of the eruption and are similar to previous effusive dome-forming eruptions, indicating bulk magmatic composition cannot be a major factor in the change in behaviour. Instead, the initiation and cessation of the 2010 eruption was predominantly controlled by magma flux from depth, with shallow conduit processes modulating eruptive style, as evidenced by microlites.

**Fig. 7:**
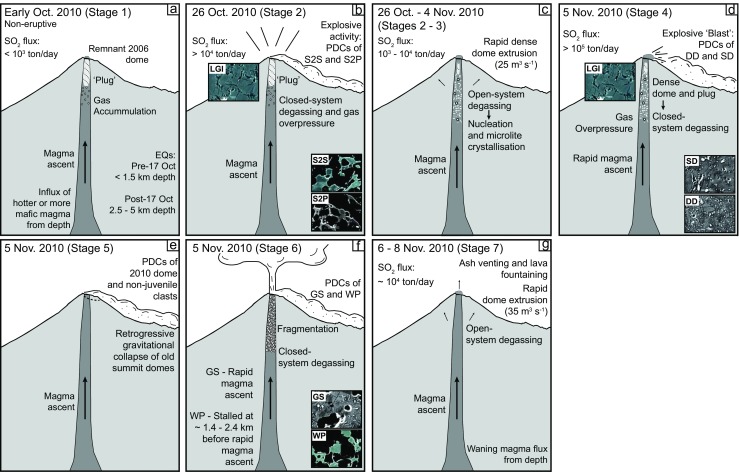
Schematic diagram of the Merapi conduit during the 2010 eruption. **a** Early October (stage 1): rapid magma ascent caused by input of hotter or more mafic magma, with the presence of a ‘plug’ in the conduit, causing gas overpressure. **b** This resulted in the initial explosions beginning on 26 October (stage 2), which opened the system, allowing for rapid dome extrusion between 26 October and 4 November (stage 3). **c** Fast magma ascent and open-system degassing promoted rapid nucleation and crystallisation with permeable gas loss during rapid dome extrusion (stage 3). **d** Permeable gas loss, vesicle collapse and formation of a dense dome, effectively sealed the conduit, producing conditions favourable to explosive activity on 5 November (stage 4) followed by **e** retrogressive gravitational collapse of old domes at the summit (stage 5). **f** Closed-system degassing sustained explosive behaviour, generating a convective column, which collapsed to produce the scoria- and pumice-rich PDCs on 5 November (stage 6). **g** A return to open-system degassing was marked by effusive activity and Strombolian jetting of gas and tephra between 6 and 8 November (stage 7). Lithology abbreviations as in Table 1

The initial explosion at the onset of the 2010 eruption on 26 October was not preceded by dome extrusion, as was the case for the 5 November paroxysmal explosions. On 26 October, it is probable that there was an influx of hotter, potentially more mafic magma (Fig.7a), as evidenced by high-An microlites in early products, as well as other petrological indications (e.g. Costa et al. 2013; Preece et al. 2014). Gas accumulation due to magmatic influx and rapid ascent (evidenced by low *φ* and *N*_A_ in initial explosive products) was not balanced by release through permeable conduit walls and fractures to the surface, leading to a build-up of overpressure in a closed system (Fig. 7a, b). The gas-charged, rapidly risen magma erupted as stage 2 scoria and pumice (Komorowski et al. 2013; Preece 2014; Drignon et al. 2014). In addition, the light grey dense material is interpreted to be a plug of cooled rigid magma that resided within the shallow system before being partially reheated and remobilised during eruption (Preece 2014; Gertisser et al. 2015). This material has an extensively crystallised groundmass, with low volumes of residual melt (glass) and abundant cristobalite. Cristobalite is often precipitated within vesicles via vapour transport in the uppermost conduit, and the extent of cristobalite mineralisation may reduce porosity and permeability (Horwell et al. 2013). It is therefore possible that cristobalite crystallisation within the light grey dense inclusion material contributed to sealing gas escape pathways prior to the 2010 eruption, augmenting gas overpressure. The remnant 2006 dome may also have been a factor in sealing gas escape pathways at the onset of eruption in 2010 (Fig. 7a). Seismic features support the fact that explosions during this initial stage of the eruption were driven by gas pressure in the conduit (Jousset et al. 2013). Volcano tectonic (VT), multi-phase (MP) and very long period (VLP) earthquakes, as well as unprecedented rates of summit deformation, were all detected prior to the eruption, attributed to fluid movement and pressure build-up in the conduit (Surono et al. 2012; Jousset et al. 2013; Budi-Santoso et al. 2013).

Once the juvenile magma had disrupted the ‘plug’ during stage 2 (Fig. 7b), rapid extrusion of a lava dome became possible (Fig. 7c). Rapid magma ascent and extrusion of a dense dome, evidenced by microlite textures and the presence of amphiboles without breakdown rims in stage 4 products, as well as by monitoring data (Surono et al. 2012; Pallister et al. 2013), are interpreted to be a contributing factor to the paroxysmal behaviour in 2010. Rapid magma ascent and degassing resulted in high degrees of *ΔT*, causing a rapid nucleation event and nucleation-dominated crystallisation of many microlites at shallow levels within the conduit (leading to high *N*_A_ values for stage 4 dome samples) (Fig. 7c). It is probable that degassing and crystallisation caused rheological stiffening of magma and increased viscosity (e.g. Sparks 1997; Melnik and Sparks 1999, 2005). This could have resulted in increased gas overpressure, if the pressure increase due to crystallisation could not be balanced via gas flow and escape to the exterior (Sparks 1997). Rapid nucleation and crystallisation concentrated the remaining gas into smaller amounts of residual melt, so that more gas bubbles formed as a result of supersaturation, promoting higher levels of bubble connectivity (Sparks 1997; Clarke et al. 2007). High bubble connectivity promotes permeable gas loss, resulting in vesicle collapse and formation of a dense lava dome (stage 4 dense dome samples) (Fig. 7d), which acted to effectively seal the conduit, forming conditions favourable to short-lived, directed dome explosions driven by conduit processes akin to Vulcanian-like explosions (Clarke et al. 2007) (Fig. 7d). Open-system degassing and crystallisation of the dense dome therefore effectively sealed the conduit, which, when combined with high magma flux rates in the conduit, led to an increase in pressurisation before the 5 November paroxysmal explosions (Fig. 7d). Sealing of the conduit is also indicated by the decrease in SO_2_ emissions during rapid dome growth at the beginning of November, compared to the explosive stages of the eruption (Surono et al. 2012).

Effective sealing of the conduit by the dome consequently ‘closed’ the system, so that magma ascending below the dome proceeded to degas, with little or no gas escape from the system (Fig. 7d). The light grey ‘plug’ material may also have contributed to this, as it is found as inclusions within the dense dome lava and as prismatically jointed clasts within the stage 4 PDC deposit. Stage 4 explosions and stage 5 retrogressive summit collapse (Fig. 7e), acted to open the system prior to stage 6. Evidence of closed-system degassing is seen in melt inclusions from stage 6 grey scoria and white pumice clasts (Preece et al. 2014), probably linked to fast magma ascent rates. Closed-system degassing and fragmentation of the magma sustained explosive behaviour, generating a pulsating subplinian convective column, which collapsed to produce the scoria- and pumice-rich PDCs during stage 6 of the 2010 eruption (Fig. 7f). The cataclysmic stages of the eruption on 5 November therefore started with repeated short directed explosions (‘blasts’), driven by conduit conditions similar to Vulcanian eruptions (stage 4), before a subplinian convective column was generated by the rising magma in an open conduit (stage 6). The following day, on 6 November, effusive activity returned, with even higher dome extrusion rates than previously (35 m^3^ s^−1^), accompanied by Strombolian (i.e. open-vent) jetting of gas and tephra in the morning of 6 November (Surono et al. 2012; Pallister et al. 2013) (Fig. 7g). This stage represents the transition from explosive (Vulcanian/subplinian) activity back to effusive activity and a return to open-system degassing. Presumably, the rapid dome extrusion that occurred on 6 November could potentially have caused further large explosions by sealing the conduit as occurred during the paroxysmal stage. Instead, observed Strombolian-style gas and tephra fountaining suggests that the preceding stage 6 explosive activity ‘re-opened’ the system, creating a pathway for gas release. Dome growth ceased by 8 November and was followed by dome subsidence and small gas emissions (Surono et al. 2012; Pallister et al. 2013). This indicates that waning magmatic flux inhibited further explosive behaviour.

## Conclusions

In summary, the beginning and end of the 2010 eruption were controlled by magma flux from depth, but eruptive style and transitions between explosive and effusive activity were regulated by shallow conduit processes. Degassing and degassing-induced crystallisation were controlled by magma ascent processes in the conduit, which in turn were responsible for changes in eruptive style. A large influx of hotter, possibly mafic magma from depth, triggered gas overpressure and faster magma ascent rates compared to those observed in 2006. The presence of a ‘plug’ in the conduit, coupled with high ascent rates led to closed-system conditions, which pressurised the system and led to explosions on 26 October. Initial explosions temporarily ‘opened’ the system, allowing ascending magma to degas more freely, and extrude as a lava dome, representing the transition back to effusive activity. Open-system degassing led to a rapid nucleation and crystallisation event and extrusion of dense, degassed lava. This effectively resealed the system, and closed-system degassing of the ascending magma began to prevail, leading to a build-up of gas overpressure until the cataclysmic dome explosions and subplinian stage on 5 November. These explosions then enabled open-conduit conditions, facilitating the transition back to effusive activity, allowing for rapid extrusion and emplacement of a dome the following day. By this stage, the eruption was waning and so further explosive activity did not occur due to decreasing magma flux from depth.

Transitions between explosive and effusive activity in 2010 were driven primarily by the dynamics of magma ascent in the conduit, with degassing and crystallisation acting via feedback mechanisms, resulting in cycles of effusive and explosive activity. For example, explosive activity on 26 October and 5 November acted to ‘open’ the system and allow for efficient degassing, causing the transition to effusive activity. Effusive activity at the beginning of November, promoted the transition back to explosive activity, with rapid dome growth effectively sealing the system. In other words, explosive activity enabled subsequent effusive activity, and effusive activity provoked explosive activity.

The 2010 eruption demonstrates the capacity for dome-forming periods at Merapi to switch to explosive behaviour and that future eruptions may begin explosively, with little warning time and without initial dome growth. Petrological evidence suggests that pre-cursors to a large eruption in the future may include a deep pulse of gas-rich magma and a build-up of gas overpressure or rapid dome extrusion. Warning signs that these processes are occurring may be monitored remotely via seismic networks, satellite monitoring, gas flux and ground deformation measurements. The findings of this study are pertinent to other dome-forming volcanoes, where the dynamics of magma influx, magma ascent, degassing and crystallisation may also play an important role in determining eruptive style.

## Electronic supplementary material

Below is the link to the electronic supplementary material.

ESM 1 (PDF 1879 kb)

ESM 2 (PDF 104 kb)

ESM 3 (PDF 691 kb)

ESM 4 (XLS 100 kb)
