# Supplementary figures and images for: Transitions between explosive and effusive phases during the cataclysmic 2010 eruption of Merapi volcano, Java, Indonesia
Source: Bull Volcanol. 2016 Jul 18;78(8):54. doi: 10.1007/s00445-016-1046-z (PMC7175738; doi:10.1007/s00445-016-1046-z)

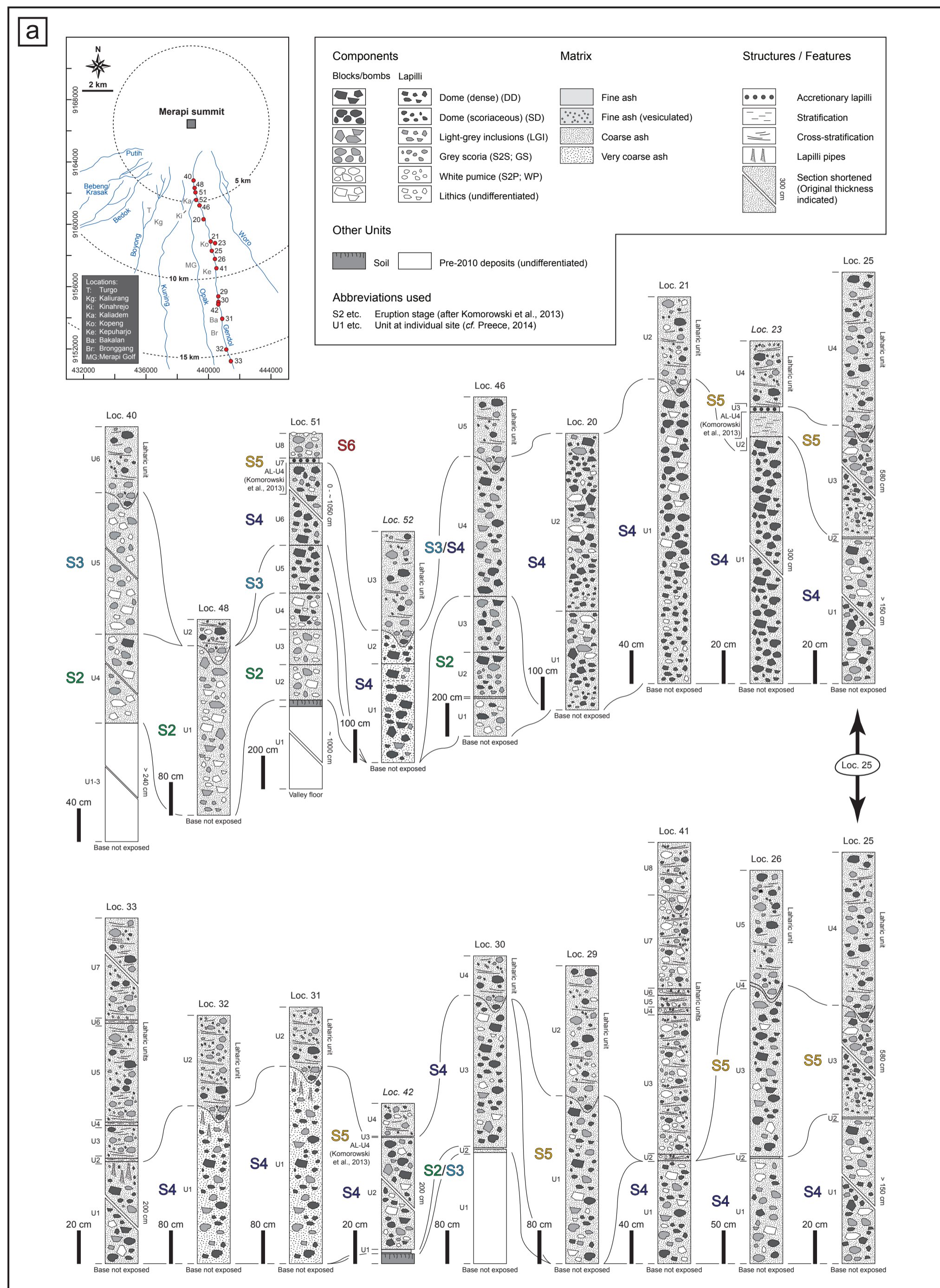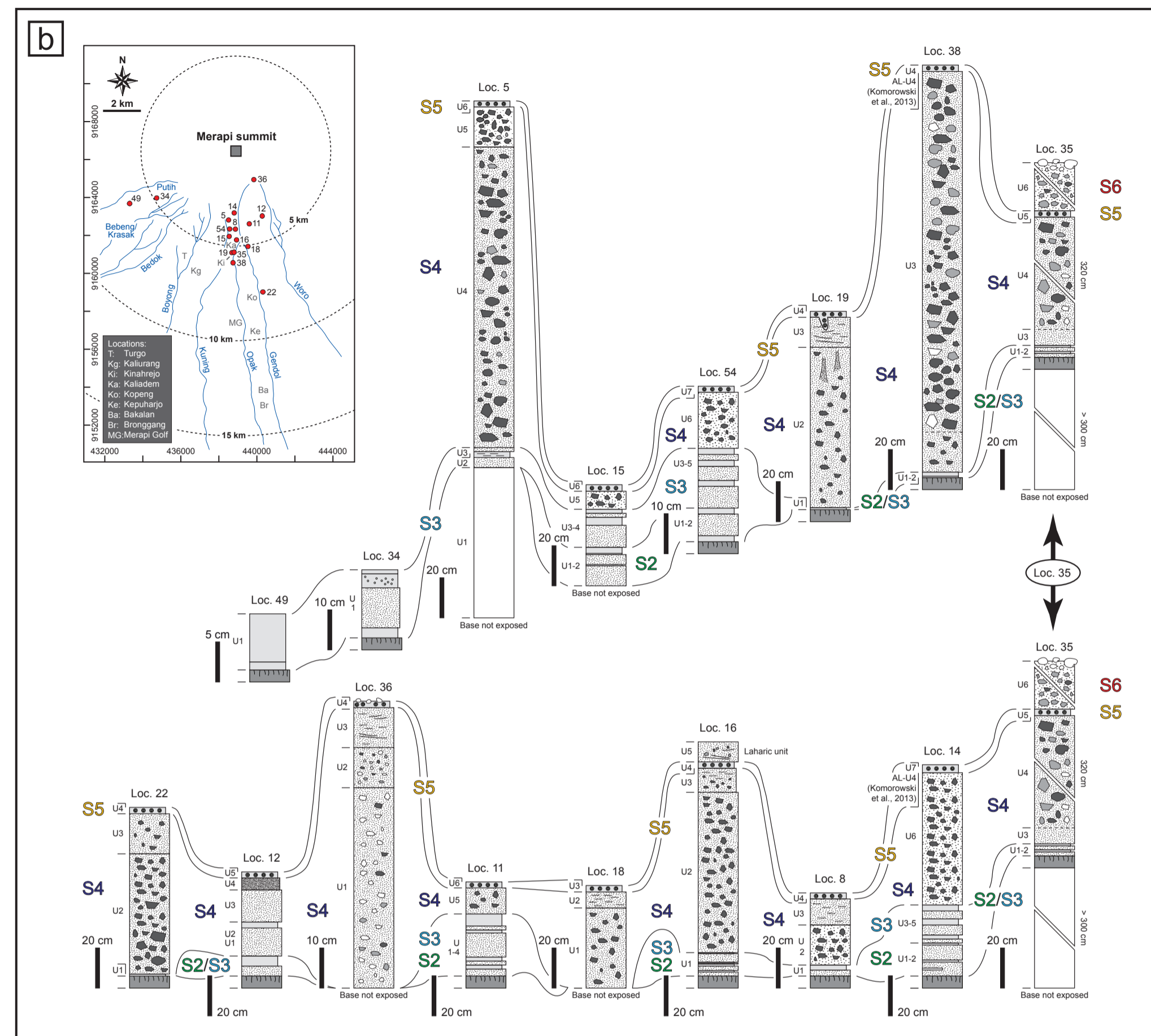

Supplement: Supplementary file 1 — (PDF 1879 kb) [file 445_2016_1046_MOESM1_ESM.pdf]
