# Supplementary material for: Transitions between explosive and effusive phases during the cataclysmic 2010 eruption of Merapi volcano, Java, Indonesia
Source: Bull Volcanol. 2016 Jul 18;78(8):54. doi: 10.1007/s00445-016-1046-z (PMC7175738; doi:10.1007/s00445-016-1046-z)
Supplement: Supplementary file 2 — (PDF 104 kb) [file 445_2016_1046_MOESM2_ESM.pdf]

Online Resource 2

2010 samples analysed in this study for whole rock geochemistry (XRF), mineral and glass compositions (EMPA) and microlite textural analysis (Textural)

Sample	Lith.*	XRF	EMPA	Textural
M11-28a	S2S		x	x
M13-26a	S2P		x	x
M13-26b	S2P		x	
M13-44	S2P		x	x
DD10	DD		x	
M11-01	DD	x		x
M11-04	DD	x		
M11-07	DD		x	
M11-10b	DD	x		
M11-12	DD	x		
M11-15	DD	x		x
M11-19a	DD	x		
M11-20	DD	x		
M11-24	DD	x	x	
M11-26a	DD	x	x	
M11-27-2	DD		x	
M11-27-3	DD		x	
M11-27-5	DD	x		
M11-38	DD		x	
M11-46	DD	x		
M11-48	DD		x	
M11-53-B1	DD	x		
M11-65	DD	x		
M11-80	DD	x		
M11-87	DD	x		
M11-95	DD	x		
M11-100	DD	x		
M11-02	SD			x
M11-06	SD	x		
M11-51	GS	x	x	x
M11-75	GS	x	x	x
M11-136b	GS	x		
M11-138a	GS	x		
M11-18	GS	x		
M11-33	WP	x	x	
M11-50	WP	x	x	x
M11-55	WP	x		x
M11-61	WP	x	x	
M11-05	LGI	x		
M11-27-2	LGI		x	
M11-27-3	LGI		x	
M11-27-4	LGI		x	
M11-38	LGI		x	
M11-48	LGI		x	
M11-96	LGI	x		
M11-103	LGI	x		
M11-104	LGI	x		
M11-130	LGI	x		
M11-131	LGI		x	
M11-135	LGI	x		
M11-136c	LGI	x		

* Lithology types: S2S = Stage 2 Scoria, S2P = Stage 2 Pumice, DD = Dome (dense), SD = Dome (scoriaceous), GS = Grey Scoria, WP = White Pumice, LGI = Light grey inclusions
