# Supplementary material for: Transitions between explosive and effusive phases during the cataclysmic 2010 eruption of Merapi volcano, Java, Indonesia
Source: Bull Volcanol. 2016 Jul 18;78(8):54. doi: 10.1007/s00445-016-1046-z (PMC7175738; doi:10.1007/s00445-016-1046-z)

Online Resource 3

Whole rock geochemical data and TAS diagram of 2010 eruptive products

Sample	Lith.*	SiO_2 (%)	TiO_2 (%)	Al_2O_3 (%)	Fe_2O_3 (%)	MnO (%)	MgO (%)	CaO (%)	Na_2O (%)	K_2O (%)	P_2O_5 (%)	LOI (%)	Total (%)
M11-01	DD	54.04	0.74	19.14	7.73	0.19	2.49	8.22	3.84	2.05	0.31	-0.14	98.61
M11-04	DD	55.40	0.73	19.12	7.65	0.19	2.34	7.96	4.00	2.17	0.32	-0.06	99.82
M11-05	LGI	53.76	0.77	19.23	8.15	0.19	2.62	8.43	3.83	2.11	0.32	-0.34	99.07
M11-06	SD	54.28	0.72	18.97	7.54	0.19	2.37	8.04	3.95	2.09	0.32	0.03	98.50
M11-10b	DD	55.45	0.73	19.36	7.59	0.20	2.41	8.00	4.07	2.16	0.32	0.00	100.29
M11-12	DD	54.77	0.74	19.13	7.79	0.20	2.42	8.13	3.80	2.08	0.32	-0.15	99.23
M11-15	DD	54.34	0.74	19.19	7.86	0.19	2.46	8.20	3.70	2.09	0.32	-0.19	98.90
M11-18	WP	55.16	0.70	19.14	7.29	0.19	2.31	7.86	3.86	2.18	0.30	0.03	99.02
M11-19a	DD	55.07	0.72	19.13	7.52	0.20	2.34	7.91	3.87	2.14	0.32	-0.09	99.13
M11-20	DD	54.73	0.72	18.93	7.56	0.19	2.37	7.86	3.83	2.13	0.32	-0.10	98.54
M11-24	DD	55.00	0.73	19.11	7.61	0.20	2.37	7.95	3.91	2.14	0.32	-0.11	99.23
M11-26a	DD	55.19	0.73	19.15	7.64	0.20	2.38	7.99	3.86	2.13	0.32	-0.07	99.52
M11-27-5	DD	54.80	0.72	19.06	7.56	0.19	2.34	7.90	3.85	2.13	0.32	-0.06	98.81
M11-33	WP	54.69	0.71	18.90	7.57	0.19	2.28	7.72	3.82	2.17	0.31	0.53	98.89
M11-46	DD	54.22	0.74	19.09	7.83	0.19	2.47	8.18	3.83	2.05	0.32	0.20	99.12
M11-50	WP	54.98	0.72	19.16	7.46	0.19	2.29	7.87	3.87	2.14	0.31	0.22	99.21
M11-51	GS	54.94	0.71	19.00	7.46	0.19	2.34	7.79	3.88	2.17	0.31	0.00	98.79
M11-53-B1	DD	54.78	0.73	18.87	7.68	0.20	2.39	7.96	3.82	2.11	0.32	0.01	98.87
M11-55	WP	54.93	0.71	18.91	7.49	0.19	2.36	7.85	3.79	2.15	0.31	0.30	98.99
M11-61	WP	55.03	0.70	19.04	7.35	0.19	2.31	7.85	3.84	2.15	0.30	0.25	99.01
M11-65	DD	54.70	0.72	18.94	7.56	0.20	2.37	7.93	3.86	2.10	0.32	0.07	98.77
M11-75	GS	55.04	0.71	19.02	7.39	0.19	2.32	7.82	3.93	2.16	0.32	0.20	99.10
M11-80	DD	54.87	0.71	18.98	7.41	0.19	2.31	7.86	3.89	2.12	0.32	0.02	98.68
M11-87	DD	54.36	0.74	18.99	7.73	0.20	2.43	8.15	3.84	2.05	0.32	0.11	98.92
M11-95	DD	55.02	0.72	19.03	7.44	0.19	2.31	7.90	3.89	2.12	0.32	-0.03	98.91
M11-96	LGI	53.34	0.79	19.20	8.36	0.19	2.74	8.62	3.64	2.01	0.31	-0.35	98.85
M11-100	DD	54.92	0.71	19.03	7.48	0.19	2.35	7.94	3.92	2.12	0.32	-0.02	98.96
M11-103	LGI	53.77	0.78	18.99	8.28	0.20	2.74	8.46	3.69	2.06	0.31	-0.33	98.95
M11-104	LGI	53.69	0.76	19.04	8.12	0.20	2.65	8.41	3.69	2.06	0.31	-0.35	98.58
M11-130	LGI	53.38	0.79	19.14	8.48	0.20	2.80	8.70	3.62	2.00	0.30	-0.26	99.15
M11-135	LGI	51.98	0.85	19.01	9.07	0.20	3.00	8.85	3.52	2.00	0.31	-0.21	98.58
M11-136b	GS	54.46	0.73	18.90	7.78	0.19	2.47	8.09	3.80	2.13	0.31	0.04	98.90
M11-136c	LGI	53.94	0.76	19.20	8.03	0.19	2.56	8.34	3.78	2.09	0.30	-0.32	98.87
M11-138a	GS	54.47	0.72	18.89	7.66	0.19	2.40	8.02	3.79	2.12	0.29	0.58	99.13

* Lithology types: DD = Dome (dense), SD = Dome (scoriaceous), GS = Grey Scoria, WP = White Pumice, LGI = Light grey inclusions

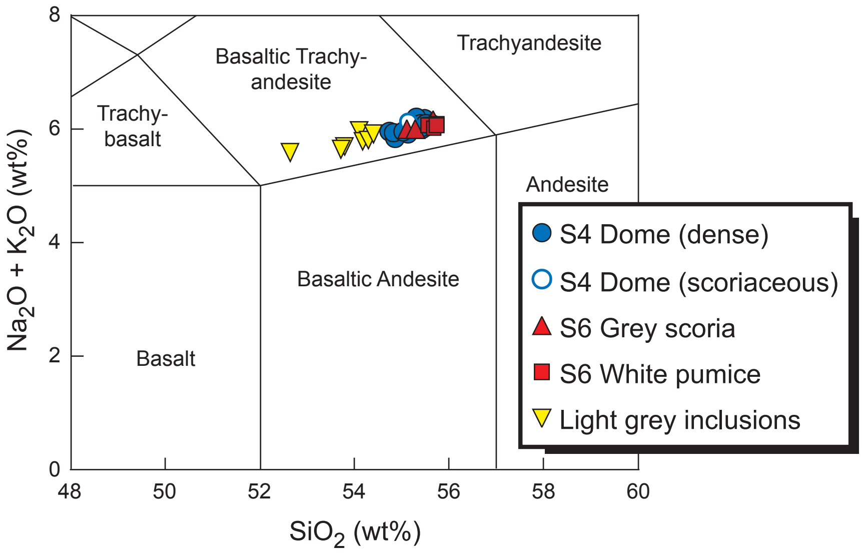

Supplement: Supplementary file 3 — (PDF 691 kb) [file 445_2016_1046_MOESM3_ESM.pdf]
